# Nutritional potentialities of some tree leaves based on polyphenols and rumen *in vitro* gas production

**DOI:** 10.14202/vetworld.2018.1479-1485

**Published:** 2018-10-23

**Authors:** K. S. Giridhar, T. M. Prabhu, K. Chandrapal Singh, V. Nagabhushan, T. Thirumalesh, Y. B. Rajeshwari, B. C. Umashankar

**Affiliations:** 1 Department of Animal Nutrition, Veterinary College, Gadag, Karnataka, India; 2 Department of Animal Nutrition, Veterinary College, Bengaluru, Karnataka, India; 3 Department of Animal Nutrition, Veterinary College, Shivamogga, Karnataka, India; 4 Department of LFC, Veterinary College, Shivamogga, Karnataka, India; 5 Department of LPM, Veterinary College, Bengaluru, Karnataka, India

**Keywords:** chemical composition, *in vitro*, *in vitro* dry matter digestibility, *in vitro* digestible organic matter, metabolizable energy, polyethylene glycol, rumen *in vitro* incubation and gas production, ruminants, tannins, tree leaves

## Abstract

**Aim:**

The study was conducted to evaluate eight tree leaves based on polyphenolic content and rumen *in vitro* incubation and gas production technique (RIVIGPT) for their nutritive potentiality.

**Materials and Methods:**

Eight selected tree leaves, namely *Sesbania grandiflora*, *Melia dubia*, *Dillenia* spp., *Artocarpus heterophyllus*, *Commiphora caudata*, *Moringa oleifera*, *Leucaena leucocephala*, and *Acacia auriculiformis*, were selected for proximate composition, forage fiber fractions, total phenolics (TPs), non-tannin phenols (NTPs), total tannins (TTs), condensed tannins (CTs), and hydrolysable tannins (HTs); RIVIGP with and without polyethylene glycol (PEG); and *in vitro* dry matter digestibility (IVDMD) (modified *in vitro* two stage) analysis was conducted. On the basis of RIVIGPT, the *in vitro* digestible organic matter (IVDOM) and dry matter intake (DMI) was calculated.

**Results:**

Crude protein (CP) content of tree leaves ranged from 9.59 to 25.81%, neutral detergent fiber (NDF) 28.16 to 53.33%, acid detergent fiber (ADF) 21.26 to 41.7%, acid detergent lignin (ADL) 3.62 to 21.98%, TP 1.83 to 17.35%, TT 0.40 to 15.47%, and CTs 0.02 to 15.26%. IVDMD (%) was ranged from 64.95 to 88.12. The mean metabolizable energy (ME) (MJ/Kg) of tree leaves estimated with and without PEG was 7.75±0.56 and 8.75±0.39, *in vitro* gas production at 24 h (IVGP^24^) (ml) 31.06±4.14 and 37.09±2.64, initial gas production (a) (ml) 0.49±0.63 and 1.33±0.72, potential gas production (D) (ml) 38.74±4.27 and 43.79±2.44, rate of gas production (*k)* (h^−1^) 0.11±0.02 and 0.11±0.013, *t_1/2_*(ml) 9.81±2.41 and 7.42±0.80, *in vitro* gas production at 96 h IVGP^96^ (ml) 39.50±4.430 and 45.14±2.65, the predicted IVDOM (%) 55.44±4.15 and 61.98±3.03, and DMI (g/Kg W^0.75^) 103.1±14.76 and 104.3±10.16, respectively. The addition of PEG showed an improvement in IVGP^24^, IVGP^96^, ME, predicted IVDOM, and predicted DMI. CP was positively correlated with ME, IVGP^24^, IVGP^96^, *a+b*, *k* (r=0.749, p<0.05), IVDMD, IVDOM, and DMI (r=0.838, p<0.05) and negatively correlated with *a* and t_1/2_. NDF, ADF, and ADL contents were negatively correlated with ME (r=0.899, p<0.05), IVGP^24^ (r=−0.867, p<0.05), IVGP^96^ (r=−0.858, p<0.05), *a+b* (p<0.05), *k* (r=−0.828, p<0.05), IVDMD, IVDOM (r=−0.853, p<0.05), and DMI and positively correlated with *a* and *t_1/2_*. TP, TT, and CT were negatively correlated with ME, IVGP, IVGP^96^, *a+b*, *k*, IVDMD, IVDOM, and DMI and positively correlated with *a* (r=0.808, p<0.05) and t_1/2_. ME (MJ/Kg) was positively correlated with IVGP^24^ (r=0.938, p<0.05), IVGP^96^ (r=0.875, p<0.05), *a+b* (r=0.813, p<0.05), *k* (r=0.731, p<0.05), IVDMD, IVDOM (r=0.985, p<0.05), and DMI (r=0.727, p<0.05) and negatively correlated with *a* and t_1/2_.

**Conclusion:**

In the present study, the potentiality of tree leaves was assessed based on CP, ADF, ADL, TP, CT, IVGP, ME, IVDMD, predicted IVDOM, and predicted DMI. Based on this, it can be concluded that *S. grandiflora, M. dubia, M. Oleifera*, and *L. leucocephala* were graded as best; *A. heterophyllus* and *C. caudata* as moderate; and *Dillenia* spp. and *A. auriculiformis* as lowest potential ruminant feed.

## Introduction

The inadequacy of nutrients is a major limitation for livestock as a deficit of 728 mT (64%) of green fodder and 157 mT (25%) of dry fodder with 27% of crude protein (CP) and 24% of total digestible nutrients in India which is expected by 2020 [1]. This can partly be overcome by feeding tree leaves, as huge quantity of biomass is available from fodder trees, which provide nitrogen, energy, minerals and vitamins and additionally have laxative effect and reduce cost of feeding [2].

Suitability of inclusion in the feeding can be assessed either by *in situ* or rumen *in vitro* incubation and gas production techniques (RIVIGPT), which would be complementary to traditional chemical measurements [3]. In comparison to *in viv*o feeding trial, the RIVIGPT is simple, less expensive, less time consuming and allow more control over experiment. As being more efficient than other *in vitro* techniques, it is suggested for determining the nutritive value of feeds containing anti-nutritive factors and for evaluating the microbial fermentation of ruminant feeds and its impact on fermentation products [4,5].

Therefore, the present study was conducted to evaluate nutritive potentialities of eight tree leaves such as *Sesbania grandiflora*, *Melia dubia*, *Dillenia* spp., *Artocarpus heterophyllus*, *Commiphora caudata*, *Moringa oleifera*, *Leucaena leucocephala*, and *Acacia auriculiformis* of different parts of Karnataka state based on polyphenolic content and RIVIGPT. The selected tree leaves are grown in this region and used in animal feeding. Except *S. grandiflora, A. heterophyllus, M. Oleifera*, and *L. leucocephala*, other tree leaves are new to investigation and there is no literature with regard to nutritive value, and hence, this study will be useful in comparing among the tree leaves.

## Materials and Methods

### Ethical approval

The Institutional Animal Ethics Committee approved to collect rumen fluid from the cannulated cow.

### Collection of tree leaves and processing

Tree leaves of *S. grandiflora* (Agase, *Kan*.), *M. dubia* (Hebbevu, *Kan*.), *Dillenia* spp. (Kaadu kanigalu, *Kan*.), *A. heterophyllus* (Halasu, *Kan*.), *C. caudata* (Konda Maavu, *Kan*.), *M. oleifera* (Nugge, *Kan*.), *L. leucocephala* (Subabul), and *A. auriculiformis* (Acacia) were chosen from different parts of Karnataka state. About 2-3 Kg of tree leaves from the single source were hand plucked, oven dried at 55°C for 48 h, and grounded to pass through size of 1 mm sieve for further analysis (*Kan*. regional language, Kannada).

### Determination of nutritive components and polyphenolic fractions

These selected tree leaves were analyzed in triplicates for proximate composition, forage fiber fractions [5], total phenolics (TPs), non-tannin phenols (NTPs), total tannin phenols (TTPs), condensed tannins (CTs), and hydrolysable tannins (HTs) [4]; RIVIGP with and without polyethylene glycol (PEG) [6]; *in vitro* dry matter digestibility (IVDMD) (modified *in vitro* two stage) [7]; and prediction of *in vitro* digestible organic matter (IVDOM) and dry matter intake (DMI) [8].

### RIVIGP

The selected tree leaves were subjected to RIVIGP (with and without PEG) for estimating metabolizable energy (ME) and rate of gas production. A lactating dairy cow producing 3 Kg of milk per day, fitted with a flexible rumen cannula of large diameter (Bar Diamond Inc., USA), receiving a basal diet consisting of finger millet straw and CFM (maize 60%, WB 35%, mineral mixture 2%, urea 2%, and salt 1%) was used as donor cow for rumen fluid. For RIVIGPT, rumen fluid was collected before offering CFM.

Feed samples (200±10 mg) were incubated with and without PEG in 100 ml calibrated glass syringes in triplicate with 30 ml mixed rumen suspension with three blank incubations and standards [6]. For PEG treatment, PEG is added twice the amount of feed sample, and blank samples with PEG were also incubated for estimating corrected gas production. Cumulative gas production was recorded after 2, 4, 6, 8, 12, 16, 24, 36, 48, 60, 72, and 96 h of incubation.

Data on gas production were fitted to the exponential equation *Y=a+b (1−e^−ct^)*, where *Y* (mL) was defined as gas production at time *t*, *a* (mL) was the initial gas production, *b* (mL) was the gas production during incubation, *a+b* or *D* (mL) was the potential gas production, and *c* (mL/h) was the fractional gas production.

The equations used to estimate the IVDOM and ME [5] are as follows:

ME (MJ/Kg)=2.2+0.1357 GP+0.0057 CP +0.0002859 EE^2^

IVDOM (%)=14.88+0.8893 GP+0.0448 CP +0.0651 TA

Where

GP=Corrected net gas production, ml/200 mg DM.

CP=Crude protein, g/Kg DM.

EE=Ether extract g/Kg DM.

TA=Total ash, g/Kg DM.

Predicted daily intake (DMI) (g DM/Kg W^0.75^) [8] was estimated using the following equation:

DMI=18.9-0.23 (a+b)+687(c)+0.11CP(g/Kg DM)

Where *a* (mL) was the initial gas production, *b* (mL) was the gas production during incubation, and *c* (mL/h) was the fractional gas production.

### IVDMD

The IVDMD was carried out using Ankom^200^ Fiber Analyzer where F57 Ankom Filter Bag (porosity: 25 µ) was used for extraction. Bags were made up of N-free Monofilament Polyester Screen Printing Fabrics.

About 400mg of dry forage samples (2 mm) were weighed into F57 Ankom Filter Bags and subjected for 48 h incubation in Mold’s buffer/rumen fluid mixture in sealed Erlenmeyer flasks followed by treatment with NDS. The dry residues were weighed, and digestibility was calculated using the following equation [7,9].


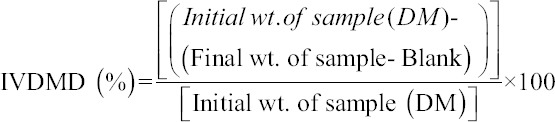


### Polyphenolic fractions

TPs were estimated by Folin–Ciocalteu reaction [4] using gallic acid as a standard. For the CT fraction, the extract was treated with butanol–HCl in the presence of ferric ammonium sulfate, and CT expressed as leukocyanidin equivalent as *A*550 nm×782.6 weight of sample DM, where *A*550 nm is absorbance at 550 nm assuming that the effective *E*1%, 1 cm, 550 nm of leukocyanidin is 460. The phenolic content of the supernatant after precipitating with polyvinylpolypyrrolidone (100 mg) was measured by the Folin–Ciocalteu reaction and this was regarded as the NTP. TTPs were calculated as the difference between TP and NTP. HTs were calculated as the difference between TTP and CT.

### Statistical analysis

The data on RIVIGP were subjected to nonlinear regression using GraphPad Prism software to assess gas kinetics (exponential decay equation model). Pearson correlation analysis was used to assess the relationship between chemical composition and ME, IVGP^24^ (*in vitro* gas production at 24 h), IVGP^96^ (*in vitro* gas production at 96 h), IVDMD, IVDOM, and DMI of the tree leaves.

## Results

### Chemical composition

The nutrient composition of the tree leaf samples is presented in Table-1. The DM content of fresh tree leaves was low in *S. grandiflora* (20.31%) and high in *A. heterophyllus* (37.45%). The OM content was low in *C. caudata* (87.97%) and high in *A. auriculiformis* (93.95%). The CP content ranged from 9.59% (*Dillenia* spp.) to 25.81% (*L. leucocephala*). The level of NDF ranged from 28.16% (*L. leucocephala*) to 53.33% (*Dillenia* spp.). The ADF ranged from 21.26% (*S. grandiflora*) to 41.7% (*Dillenia* spp.). The level of ADL ranged from 3.62% (*S. grandiflora*) to 21.98% (*A. auriculiformis*).

**Table-1:** Chemical composition^1^ (% DMB) of tree leaves.

Tree leaves	DM	OM	CP	EE	TA	AIA	NDF	ADF	ADL	ADF ash
*Sesbania grandiflora*	20.31±0.62	91.27±0.39	24.27±0.23	4.42±0.01	8.73±0.39	0.2±0.32	30.26±0.19	21.26±1.07	3.62±0.17	0.27±0.22
*Melia dubia*	34.12±0.84	89.91±0.16	16.50±0.20	4.04±0.02	10.09±0.16	0.17±0.23	30.82±0.18	22.53±0.18	8.25±1.08	0.03±0.08
*Dillenia* spp.	31.0±1.53	90.14±0.11	9.59±0.10	2.34±0.07	9.86±0.11	2.93±0.09	53.33±1.02	41.7±0.99	15.25±0.05	4.20±1.03
*Artocarpus heterophyllus*	37.45±0.74	88.97±0.54	13.16±0.18	4.96±0.07	11.03±0.54	4.56±0.34	43.17±0.82	36.41±0.89	9.14±1.09	4.85±0.12
*Acacia auriculiformis*	34.91±0.38	93.91±0.33	13.29±0.45	5.59±0.03	6.09±0.33	0.31±0.34	45.70±0.44	40.41±0.30	21.98±0.72	0.80±0.20
*Moringa oleifera*	30.90±0.85	89.09±0.60	21.56±1.13	4.20±0.28	10.91±0.60	0.47±0.56	31.70±1.12	26.40±0.44	4.57±0.33	0.65±0.23
*Leucaena leucocephala*	34.00±2.35	92.91±0.18	25.81±0.09	3.33±0.06	7.09±0.18	0.61±0.27	28.16±0.22	21.57±0.96	8.07±0.35	0.27±0.16
*Commiphora caudata*	28.74±0.76	87.94±0.18	17.05±0.13	3.73±0.01	12.06±0.18	1.58±0.43	34.28±0.34	31.95±0.05	15.30±0.04	2.83±0.02

^1^All values were mean of triplicates. DMB=Dry matter basis, DM=Dry matter, OM=Organic matter, CP=Crude protein, EE=Ether extract, TA=Total ash, AIA=Acid Insoluble Ash, NDF=Neutral detergent fiber, ADF=Acid detergent fiber, ADL=Acid detergent lignin

### Polyphenolic fractions

Polyphenolic fractions (on DM basis) of the eight tree leaves are presented in Table-2 [4]. The TP content of tree leaves was high in *A. heterophyllus* (17.35%) and low in *S. grandiflora* (1.77%). The NTP was rich in *Dillenia* spp. (5.7%) and low in *S. grandiflora* (1.00%). TTP content was more in *A. heterophyllus* (15.47%) and low in *M. oleifera* (0.40%). The potent source of CT and HT was *A. heterophyllus* (15.26%) and *Dillenia* spp. (4.72%), respectively.

**Table-2:** Polyphenolic fractions (%) of tree leaves on DMB.

Tree leaves	TP^1^	NTP^2^	TTP^3^	CT^4^	HT^5^
*Sesbania grandiflora*	1.77±0.06	1.00±0.03	0.77±0.02	0.02±0.01	0.75±0.05
*Melia dubia*	1.89±0.11	1.11±0.02	0.77±0.06	0.19±0.02	0.58±0.03
*Dillenia* spp.	11.67±0.56	5.70±0.40	5.97±0.43	1.25±0.01	4.72±0.35
*Artocarpus heterophyllus*	17.35±0.14	1.88±0.01	15.47±0.19	15.26±0.01	0.21±0.03
*Acacia auriculiformis*	9.04±0.66	4.95±0.14	4.09±0.08	2.37±0.05	1.72±0.01
*Moringa oleifera*	1.83±0.19	1.44±0.03	0.40±0.08	0.19±0.01	0.20±0.07
*Leucaena leucocephala*	4.76±0.12	2.68±0.15	2.07±0.20	1.99±0.01	0.09±0.08
*Commiphora caudata*	4.98±0.22	3.35±0.32	1.63±0.26	1.25±0.01	0.38±0.11

All values were mean of triplicates.

1 TP by Folin–Ciocalteu reaction [4].

2 NTP by Folin–Ciocalteu reaction using PVPP [4].

3 TTP by difference between TP and NTP.

4 CT expressed as leukocyanidin equivalent.

5 HT calculated by difference between TTP and CT. TP=Total phenol, NTP=Non-tannin phenol, TTP=Total tannin phenol, CT=Condensed tannin, HT=Hydrolysable tannin, DMB=Dry matter basis

### Gas production kinetics

RIVIGP values of tree leaves with or without PEG are shown in Table-3. The IVGP^24^ (ml) was more in *M. oleifera* (42.63) and less in *A. auriculiformis* (8.68). The IVGP^96^ (ml) was higher for *S. grandiflora* (50.54) and less for *A. auriculiformis* (10.27). The *D* (ml) value was more in *S. grandiflora* (47.48) and lower for *A. auriculiformis* (9.64). *k* was higher for *M. dubia* and lower for *Dillenia* spp., and t_1/2_ (h) was highest for *Dillenia* spp. and least for *M. dubia*. Among the evaluated tree leaves, energy content (ME, MJ/Kg) was higher in *S. grandiflora* (9.87) and lower in *A. auriculiformis* (5.03).

**Table-3:** Rumen *in vitro* gas production values of tree leaves with and without PEG 6000.

Tree leaves	ME (MJ/Kg)		Corrected net gas production (ml/24 h)		Rapidly produced gas (ml) (*a*)		Potential cumulative gas production (ml) (*P*)		Rate of gas production/h (*k*)		Half-life (t_1/2_)		Corrected net gas production (ml/96 h)	

	PEG 6000													
	-	+	-	+	-	+	-	+	-	+	-	+	-	+
*Sesbania grandiflora*	9.87±0.81	10.01±1.2	42.03±1.45	42.92±0.81	−2.62±1.2	−2.74±1.3	47.48±1.01	48.05±0.65	0.11±0.27	0.12±0.25	8.84±0.31	8.47±1.22	50.54±2.22	51.24±1.06
*Melia dubia*	9.37±0.12	9.74±0.43	42.47±0.32	45.17±1.19	0.31±0.87	3.16±0.67	45.81±0.64	49.33±1.12	0.16±0.88	0.15±0.20	4.24±0.55	4.80±0.73	47.91±0.32	51.33±0.97
*Dillenia* spp.	6.21±0.32	7.76±0.12	24.37±0.54	35.81±1.08	0.64±0.11	2.16±0.34	41.39±1.63	44.73±0.14	0.03±0.12	0.07±0.14	21.42±0.68	10.49±0.78	38.87±0.52	45.64±1.05
*Artocarpus heterophyllus*	7.16±0.57	9.03±0.71	25.85±0.33	39.62±0.37	4.00±0.26	0.55±0.54	40.87±0.42	45.34±0.91	0.04±0.76	0.10±0.76	19.42±0.87	6.64±0.88	39.65±0.03	46.61±0.75
*Acacia auriculiformis*	5.03±0.87	6.70±0.89	8.68±0.49	21.02±0.85	0.17±0.32	4.11±0.42	9.64±0.65	27.33±0.86	0.14±0.81	0.06±0.71	4.98±0.78	10.84±0.71	10.27±0.51	27.73±0.62
*Moringa oleifera*	8.28±0.88	9.45±0.81	42.63±0.76	40.63±0.83	0.64±0.42	1.11±0.58	44.32±0.14	46.7±0.31	0.12±0.31	0.11±0.45	6.04±0.14	6.52±0.57	46.23±1.06	48.36±1.57
*Leucaena leucocephala*	8.08±0.62	8.54±0.75	30.15±0.69	33.54±1.50	0.8±0.98	0.98±0.16	41.12±0.64	45.21±0.11	0.14±0.51	0.17±1.65	5.81±0.19	5.35±0.18	41.23±0.58	44.37±0.73
*Commiphora caudata*	7.96±0.54	8.73±0.62	32.33±1.06	38.01±1.66	−0.02±0.11	1.34±0.31	39.25±1.45	43.62±0.12	0.08±0.17	0.11±0.58	8.85±0.27	6.24±0.43	41.26±2.43	45.85±0.85

All values were mean of triplicates. +=Indicates with PEG,-=Indicates without PEG. ME=Metabolizable energy

### IVDMD

IVDMD of tree leaves was analyzed using Ankom^200^ Fiber analyzer based on modified *in vitro* two-stage method which is shown in Table-4. IVDMD of tree leaves ranged from 64.95% (*A. auriculiformis*) to 88.12% (*M. dubia*) and an average of 76.02%.

**Table 4:** IVDMD and predicted IVDOM and DMI (g/Kg W^0.75^) of tree leaves.

Tree leaves	IVDMD^1^	IVDOM^2^ (%)		DMI^3^ (g/Kg W^0.75^)	

		PEG 6000			

		-	+	-	+
*Sesbania grandiflora*	78.93±1.11	69.05±0.41	69.84±0.42	110.2±0.21	116.3±0.39
*Melia dubia*	88.12±2.43	66.61±0.17	69.01±0.31	136.8±0.42	125.0±0.51
*Dillenia* spp.	75.92±1.43	47.27±0.42	57.44±0.18	41.9±0.17	64.6±0.28
*Artocarpus heterophyllus*	71.43±1.78	50.94±0.32	63.19±0.42	48.7±0.53	94.7±0.61
*Acacia auriculiformis*	64.95±1.86	32.52±0.19	43.50±0.38	48.1±0.42	71.2±0.51
*Moringa oleifera*	86.26±1.32	60.10±0.22	67.77±0.32	111.4±0.38	104.9±0.43
*Leucaena leucocephala*	70.62±0.64	57.87±1.21	60.88±0.19	134.0±0.52	153.7±0.58
*Commiphora caudata*	71.96±0.71	59.12±0.82	64.17±0.91	82.4±0.72	103.9±0.75

All values were mean of triplicates. ^+^Indicates with PEG and -indicates without PEG.

1 IVDMD (%) - As per modified two-stage fermentation using Ankom^200^ Fiber Bag Analyzer.

2 IVDOM (%)=14.88+0.8893 GP+0.0448 CP+0.0651 TA.

3 DMI (g DM/Kg W^0.75^)=18.9-0.23 (a+b)+687(c)+0.11CP (g/Kg DM). IVDMD=*In vitro* dry matter digestibility, IVDOM=*In vitro* digestible organic matter, PEG=Polyethylene glycol, DMI=Dry matter intake

### Prediction of IVDOM and DMI

The IVDOM and DMI were predicted based on gas production and chemical composition of tree leaves which are presented in Table-4. IVDOM was ranged from 32.52 (*A. auriculiformis*) to 69.05 (*S. grandiflora*) and the predicted DMI (g/Kg W^0.75^) was ranged from 41.9 g *Dillenia* spp. to 136.8 g (*M. dubia*).

### Effect of PEG on gas production kinetics, IVDOM, and DMI

The PEG inclusion during incubation resulted in increased ME, *a, D*, IVGP^24^, k, IVGP^96^, IVDOM, and DMI of tree leaves and decreasedt_1/2_. In *Dillenia* spp.*, A. heterophyllus*, and *A. auriculiformis* tree leaves, it was more pronounced.

### Correlation analysis (r)

Pearson correlation analysis was made to test the relationship between chemical composition and ME, IVGP^24^, IVGP^96^, IVDMD, IVDOM, and DMI values and is presented in Table-5. CP was positively correlated with ME, IVGP^24^, IVGP^96^, *a+b*, *k* (r=0.749, p<0.05), IVDMD, IVDOM, and DMI (r=0.838, p<0.05) and negatively correlated with *a* (r=−0.451) and t_1/2_ (r=−0.575). NDF, ADF, and ADL contents are negatively correlated with ME (r=−0.899, p<0.05), IVGP^24^ (r=−0.867, p<0.05), IVGP^96^ (r=−0.858, p<0.05), *a+b* (r=−0.828, p<0.05), *k* (r=−0.877, p<0.05), IVDMD (r=−0.674), IVDOM (r=−0.853, p<0.05), and DMI (r=−0.538) and positively correlated with *a* (r=0.424) and *t_1/2_* (r=0.683). TP, TTP, and CT are negative correlation of non-significant difference with ME, IVGP^24^, IVGP^96^, *a+b*, *k*, IVDMD, IVDOM, and DMI and positively correlated with *a* (r=0.808, p<0.05) and t_1/2_. The initial gas production was more in tannin-containing feeds which may due to easily degradable OM. At 96 h incubation, tannins inhibited the gas production (negative correlation) which may be due to fiber bound tannins in tree leaves. The negative relationship between chemical composition and phenolic compounds, with *in vitro* degradability of legumes at 24 h of *in vitro* incubation may be due to presence of ADF and CT in them, but on addition of PEG reversed that situation [3, 10, 11]. ME (MJ/Kg) was positively correlated with IVG^P24^ (r=0.938, p<0.05), IVG^P96^ (r=0.875, P<0.05), *(a+b)* (r=0.813, p<0.05), k (r=0.731, p<0.05), IVDMD, IVDOM (r=0.985, p<0.05), and DMI (r=0.727, p<0.05) and negatively correlated with a and t_1/2_.

**Table-5:** Correlation coefficients (r) between *in vitro* fermentation parameters, IVDMD, IVDOM, and DMI with chemical composition and total phenolics and tannins.

Parameter	PEG	CP	NDF	ADF	ADL	TP	TTP	CT	ME (MJ/Kg)
ME (MJ/Kg)	-	0.684	−0.822*	−0.899*	−0.849*	−0.673	−0.448	−0.280	-
	+	0.543	−0.700	−0.774*	−0.906*	−0.480	−0.214	−0.046	-
IVGP^24^	-	0.576	−0.722*	−0.800*	−0.867*	−0.666	−0.459	−0.327	0.938*
	+	0.275	−0.472	−0.581	−0.797*	−0.328	−0.106	−0.017	0.848*
IVGP^96^	-	0.465	−0.560	−0.678	−0.858*	−0.413	−0.218	−0.132	0.875*
	+	0.346	−0.474	−0.604	−0.824*	−0.332	−0.133	−0.063	0.844*
a	-	−0.451	0.362	0.424	0.128	0.752*	0.808*	0.826*	−0.413
	+	−0.586	0.411	0.489	0.708*	0.191	0.022	−0.066	−0.629
*a+b*	-	0.384	−0.457	−0.593	−0.828*	−0.299	−0.117	−0.058	0.801*
	+	0.368	−0.476	−0.613	−0.830*	−0.300	−0.108	−0.040	0.813*
k	-	0.402	−0.470	−0.418	0.073	−0.597	−0.602	−0.487	0.099
	+	0.749*	−0.854*	−0.877*	−0.638	−0.498	−0.320	−0.112	0.731*
t_1/2_	-	−0.575	0.683	0.570	0.069	0.766*	0.735*	0.559	−0.290
	+	−0.501	0.764*	0.705	0.587	0.366	0.156	−0.076	−0.672
IVDMD	-	0.228	−0.425	−0.548	−0.674	−0.572	−0.421	−0.378	0.691
IVDOM^2^ (%)	-	0.633	−0.774*	−0.853*	−0.841*	−0.642	−0.434	−0.287	0.985*
	+	0.487	−0.644	−0.718*	−0.872*	−0.460	−0.227	−0.091	0.921*
DMI^4^ (g/Kg W^0.75^)	-	0.494	−0.538	−0.485	0.005	−0.637	−0.629	−0.498	0.159
	+	0.838*	−0.900*	−0.906*	−0.646	−0.560	−0.387	−0.168	0.727*

* p<0.05; ^+^indicates with PEG and -indicates without PEG. CP=Crude protein, NDF=Neutral detergent fiber, ADF=Acid detergent fiber, ADL=Acid detergent lignin, TP=Total phenolic, TTPs=Total tannin phenols, CTs=Condensed tannins, ME=Metabolizable energy (MJ/Kg DM), IVGP^24^=Gas production volume (ml/0.2 g DM) after 24 h of incubation, IVGP^96^=Gas production volume (ml/0.2 g DM) after 96 h of incubation, a=Rapidly produced gas (ml), *a+b* = Potential cumulative gas production (ml), k=Rate of gas production/h, t1/2=Half-life, IVDMD=In vitro DM digestibility (g/Kg DM), IVDOM=*In vitro* digestibility of organic matter (%), DMI=Dry matter intake (g/Kg W^0.75^), PEG=Polyethylene glycol

## Discussion

### Chemical composition

In the present study, CP varied from 9.59 to 25.81%, and it was in similar range for tree leaves as in published reports [12-17]. Similarly, the NDF, ADF, and ADL contents of analyzed tree leaves were in a instead of the similar range [13,18,19].

In the present study, there were considerable variations in chemical compositions between the tree leaves. This was due to differences in genotype, environment, stage of maturity, and harvesting [20-22]. The multipurpose tree leaves contained moderate levels of CP, minerals, and vitamins that are deficient in many low-quality roughages and it was found that CP level above the threshold level (11-12%) is required for the moderate level of ruminant production [23].

### Polyphenolic fractions

The literature on the polyphenolic content of tree leaves depicts that the concentration remarkably varies from source to source. The polyphenolic content estimated was lower [17,24,25] than the present values, whereas a similar range of values was also observed in the past [15,16,26-28].

Tannin composition in plants depends on type of plant, photosynthetic capacity, soil fertility, environmental conditions, maturity of the leaves, and processing and analytical method employed in analysis [4]. In general, the intake of CT below 5% improves the utilization of feed by ruminants, mainly because of a reduction in ruminal protein degradation and, as a consequence, greater availability of mainly essential amino acids for absorption in the small intestine. Values of CTs exceeding 5% on dry matter basis could inhibit microbial activity, depress dry matter digestibility, and reduce voluntary intake [29].

### Gas production kinetics

In the present study, the lower level of ME and RIVIGP parameters is closely related to its CP, fiber fractions, and tannins as reported by previous studies [24,30, 31, 32,33].

### IVDMD and prediction of IVDOM and DMI

IVDMD of tree leaves ranged from 64.95% (*A. auriculiformis*) to 88.12% (*M. dubia*) and an average value of 76.02%, whereas in previous study, it was found the range of IVDMD in tree leaves between 22.70 and 58.72 % that was lower than the present study which may arise due to lower quality of tree leaves [19]. The predicted IVDOM ranged from 32.52 (*A. auriculiformis*) to 69.05 (*S. grandiflora*) with an average value of 55.43%, and the predicted DMI (g/Kg W^0.75^) was lowest in *Dillenia* spp. (42.2 g) with an average value of 103.13 g.

### Effect of PEG on kinetics of gas production

The improvement in gas production in *Dilleni*a spp.*, A. heterophyllus*, and *A. auriculiformi*s tree leaves was due to affinity of PEG to tannins[32-35]. It was also found that there was only minor improvement in IVGP^24^ and IVGP^96^ in *L. leucocephala* and *C. caudata* due to the low level of CT in them. Addition of PEG also increased the ME, IVDOM % content, and DMI (g/KgW^0.75^) in these tree leaves.

In general, the improvement in fermentation in each species by adding PEG almost certainly reflects its deactivation of secondary compounds [11,35,36]. High CT (proanthocyanidins) and fiber (NDF and ADF) contents reduce digestibility [31,32] while low CP affects the acceptability of browse [25]. Similarly, in the present study, lower level of IVGP was observed in those tree leaves which are containing more CT, NDF, ADF, and ADL.

Based on ME, ADL, and predicted DMI of tree leaves, it can be categorized as best, moderate and poor potential source of fodders for ruminants [26]. *S. grandiflora, M. dubia, M. oleifera*, and *L. leucocephala* were best potential fodders due to higher ME (>8.08 MJ) and predicted DMI (>111 g) and lower ADL (<8.62%); *A. heterophyllus* and *C. caudata* were moderate potential due to moderate in ME (7-7.96MJ) and predicted DMI (48.7-82.4 g) and slightly higher ADL (9-15.3%), whereas, *Dillenia* spp. and *A. auriculiformis* were lowest potentiality due to lower ME (5-6.2 MJ) and predicted DMI (41-48.1 g) and very higher ADL (15.25-21.98%) in them.

## Conclusion

In the present study, the nutritive potentiality of tree leaves was assessed based on CP, ADF, ADL, TP, CT, IVGP, ME, IVDMD, IVDOM, and DMI. It can be concluded that *S. grandiflora, M. dubia, M. oleifera*, and *L. leucocephala* were graded as the best due to higher CP, ME, IVGP, IVDMD, IVDOM and DMI and lower polyphenols and fiber fractions; *A. heterophyllus* and *C. caudat*a were graded as the moderate potential and *Dillenia* sp. and *A. auriculiformis* were graded as the lowest potential as a ruminant feed. While the addition of PEGin *Dillenia* spp. and *A. auriculiformi*s improved the RIVIGP but it did not impact their overall nutritive ranking. However, more studies are required to characterize these feeds better through *in vivo* feeding trials with respect to palatability and intake.

## Authors’ Contributions

TMP and KCS planned and supervised the entire research work. KSG carried out the experimental work and laboratory analysis. VN, TT, YBR, and BCU prepared the manuscript along with data analysis. All authors read and approved the final manuscript.
